# Hospital networks and patient transport capacity during the COVID-19 pandemic when intensive care resources become scarce

**DOI:** 10.1186/s13054-021-03462-3

**Published:** 2021-01-12

**Authors:** Alexander Supady, Dawid Staudacher, Christoph Bode, Guido Michels, Tobias Wengenmayer

**Affiliations:** 1 Department of Medicine III (Interdisciplinary Medical Intensive Care), Medical Center – University of Freiburg, Faculty of Medicine, University of Freiburg, Freiburg, Germany; 2 Department of Cardiology and Angiology I, Heart Center, University of Freiburg, Hugstetter Strasse 55, 79106 Freiburg, Germany; 3 Heidelberg Institute of Global Health, University of Heidelberg, Heidelberg, Germany; 4 Department of Acute and Emergency Care, St. Antonius Hospital Eschweiler, Eschweiler, Germany

With great interest we read the article by Heinsar et al. discussing implications for extracorporeal membrane oxygenation (ECMO) during the coronavirus disease 2019 (COVID-19) pandemic when the number of patients reaches a level that demand may overwhelm available resources [1].

While various medical resources could become scarce during a pandemic, ECMO is exposed for numerous reasons. Firstly, it is a potentially lifesaving treatment for patients that would die without; secondly, ECMO treatment is highly resource intensive and therefore may have  an impact on the availability of resources for other patients [2].

Crisis standards of care may include strategies for rationing of scarce resources [3]. However, before withholding potentially lifesaving therapy including ECMO from patients in need, all reasonable efforts must be made to maintain ordinary standard of care [4].

During the COVID-19 pandemic, disease hotspots emerged, where healthcare facilities were overwhelmed and provision of standard of care was challenged or even impossible. Examples include Wuhan (China), Bergamo (Italy), New York (United States) and Heinsberg (Germany). However, at the same time, even within these affected countries, healthcare capacities were still available.

In case of imminent local overstrain, networks could be established to transfer patients to regions with lower case numbers, whether nearby or further away (Fig. 1) [5]. Thoughtful and responsible planning should include making information about free hospital and ICU beds and other potentially scarce healthcare resources, like ventilators or ECMO, at the local, regional and national level easily available to physicians. Furthermore, governments, administrations, and healthcare managers should provide transport capacity to transfer patients from congested regions to less affected regions where there are still hospital resources available. In France, when some regions were particularly affected by the pandemic, passenger trains were temporarily converted for the transfer of intensive care patients.

**Fig. 1:**
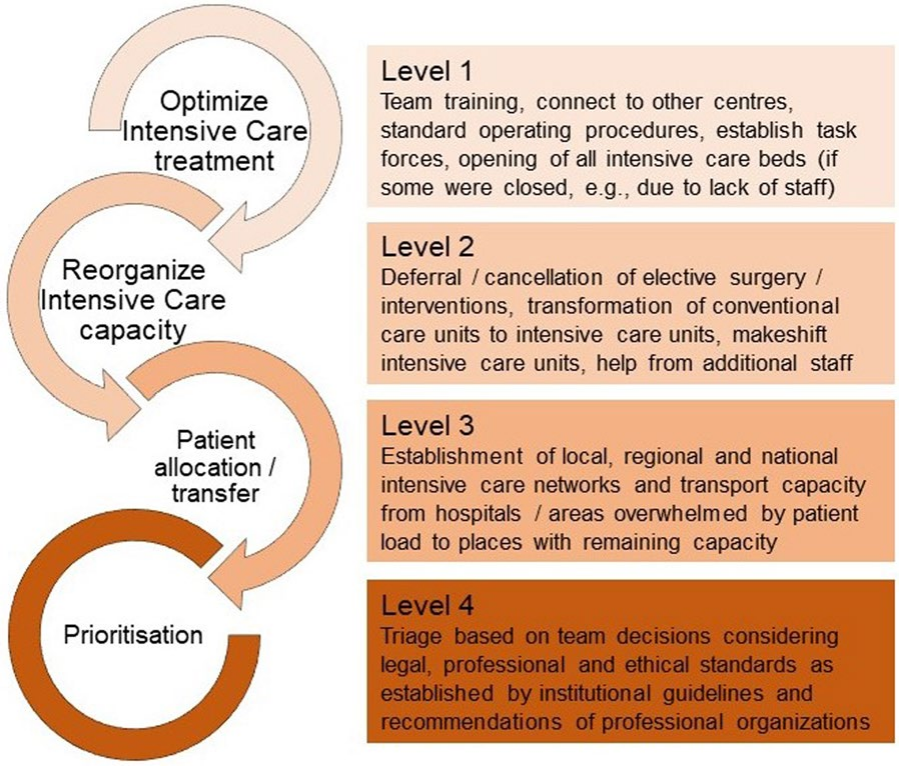
Levels for gradually moving from standard of care to crisis standard of care when intensive care unit resources become scarce during the COVID-19 pandemic

Registries monitoring the availability of ICU beds and definition of regional clusters for patient allocation help to ease pressure from overburdened hospitals, too. This model could be established elsewhere.

Triage committees or advanced prediction models may support decision makers in preparing for situations of scarcity; however, as soon as important resources are no longer available in sufficient quantities, structures and procedures must be in place that healthcare workers can access without any particular hurdles in order to refer or transfer patients to the places where the necessary resources are (still) available. In such situations triage committees or prediction models most likely cannot keep up with the pace and flexibility required.

## Data Availability

Not applicable.
